# Sociodemographic and Clinical Profile of Homeless Patients Attending the Emergency Department of a Tertiary Care Centre in Rishikesh, Uttarakhand, India: A Prospective Observational Study

**DOI:** 10.7759/cureus.116250

**Published:** 2026-09-14

**Authors:** Rinku Meena, Bharat Bhardwaj, Vempalli Nagasubramanyam, Anand M Gangdev, Devinder Kumar Lalotra, Jitendra Yadav

**Affiliations:** 1 Emergency Medicine, National Institute of Medical Sciences (NIMS) University, Jaipur, IND; 2 Emergency Medicine, All India Institute of Medical Sciences, Rishikesh, Rishikesh, IND; 3 Emergency Medicine, All India Institute of Medical Sciences, Mangalagiri, Mangalagiri, IND

**Keywords:** clinical profile, ed outcomes, emergency department, homelessness, india, sociodemographic profile, triage, vulnerable population

## Abstract

Introduction

Homeless individuals are a highly vulnerable population with complex medical and social needs. Emergency departments (EDs) often serve as the primary point of contact for healthcare for homeless persons in India, yet systematic data on their profile and outcomes from tertiary care settings remain limited.

Methodology

A prospective observational study was conducted in the emergency medicine department of a tertiary care centre in North India from July 2022 to December 2023 (18-month duration). All homeless patients aged over 18 years presenting to the emergency department and meeting inclusion criteria were enrolled. Sociodemographic data, clinical presentation, investigations, consultations and ED outcomes were recorded using a structured pro forma, and data were analysed using SPSS v23.0 (IBM Corp., Armonk, NY).

Results

Forty-eight homeless patients were enrolled in the study. The majority were men (93.8%), adults aged 31-60 years (52.1%) and from Uttarakhand (85.4%). Most patients (75%) were staying alone, and 81.2% were uninsured. The most common mode of arrival was ambulance (85.4%), with 66.7% referred from another healthcare facility. The most common presenting complaints were abdominal symptoms (22.9%), dyspnoea (18.8%) and trauma (16.7%). Chronic obstructive pulmonary disease (COPD) was the most common diagnosis (12.5%), followed by head injury (10.4%) and decompensated chronic liver disease (8.3%). Two-thirds of the patients (66.7%) were triaged to the red area. ED in-hospital mortality was 18.8%. The median ED length of stay was two days. Fourteen patients had ED revisits during the study period.

Conclusions

Homeless patients presenting to the ED are predominantly men and uninsured and arrive in critical condition via ambulance. They carry a high burden of chronic disease, present late in the course of illness and have substantially high in-ED mortality. Integrated care models, policy-level expansion of health coverage and dedicated ED protocols for homeless individuals are urgently needed in India.

## Introduction

Food, clothing and shelter are three basic human needs. While many individuals struggle to meet the first two, permanent residence often remains beyond reach for a significant proportion of the population [1]. Homelessness is a growing global phenomenon with diverse definitions across countries. The definition of homelessness according to the Census of India 2011 is 'an individual who resides in a shelter or place not meant for human habitation and who is exiting an institution where he or she temporarily resided and stays on pavements, roadsides, railway platforms, staircases, temples, streets, pipes or other open spaces' [2].

India is home to approximately 0.45 million homeless families, comprising a total homeless population of 1.773 million individuals, with the highest burden in Uttar Pradesh and Maharashtra [3]. In search of shelter and food, a homeless person attends the emergency department (ED), and due to their inability to reach the primary health centre, homeless patients have high rates of ED utilisation [4,5].

Due to difficulty accessing outpatient facilities, homeless persons are often dropped at the ED by EMS personnel, autorickshaw drivers or members of the public. They frequently present without money, identification or a caretaker. In some cases, individuals deliberately seek the ED during extreme weather conditions in search of shelter, food and basic safety.

Previous studies have shown that homeless patients frequently use emergency departments and have distinct demographic and clinical characteristics. Older homeless adults are more likely to have alcohol abuse and arrive by ambulance, while homeless patients overall have higher rates of ED revisits and hospital readmissions, particularly after discharge to shelters or the streets. Compared with non-homeless patients, they are often younger but have a greater likelihood of hospitalisation despite similar healthcare costs and lengths of stay. These findings emphasise the necessity of targeted interventions to improve the continuity of care and reduce recurrent emergency department utilisation [6-9].

Previous studies consistently demonstrate that homeless patients have higher rates of emergency department utilisation, ambulance use, substance abuse and mental health disorders than non-homeless individuals. They also experience longer ED stays and increased 30-day revisits and hospital readmissions, particularly among those with mental illness. Homeless individuals are more likely to have infectious diseases, alcohol and drug dependence, social isolation and injury-related presentations, especially involving the lower limbs. These findings emphasise the necessity of integrated medical, psychiatric and social interventions to improve outcomes in this vulnerable population [10-14].

Studies consistently show that homeless patients are more likely to arrive at the emergency department by ambulance, be uninsured and present with substance abuse, alcohol-related problems and injuries. They have significantly higher rates of emergency department revisits and 30-day hospital readmissions than non-homeless individuals. Despite lower hospital admission rates, the emergency department remains a critical point of access for healthcare among the homeless population, pointing out the need for targeted interventions and improved continuity of care [15-19].

The primary outcome of the study is the description of the sociodemographic and clinical profile of homeless patients.

Secondary outcomes of the study are (1) ED disposition (discharged, admitted to ward, admitted to ICU, died in ED, left against medical advice {LAMA} or absconded), (2) ED length of stay, (3) in-ED mortality and (4) ED revisits during the study period.

## Materials and methods

Study design and setting

A single-centre, prospective observational study was conducted at the Department of Emergency Medicine of All India Institute of Medical Sciences (AIIMS), Rishikesh, a tertiary care academic hospital located in Rishikesh, Uttarakhand, India. The study period extended from July 2022 to December 2023 (18 months total, with 15 months of data collection, one month for follow-up and two months for data analysis and report writing).

Participants

Inclusion Criteria

All homeless patients aged >18 years who presented to the ED during the study period and were willing to participate in the study were included.

Exclusion Criteria

Patients who declined to participate in the study were excluded.

Operational Definition of Homelessness

According to the Census of India, an individual is considered homeless if they reside in a shelter or a place not meant for human habitation, are exiting an institution where they temporarily resided or stay on pavements, roadsides, railway platforms, staircases, temples, streets, pipes or other open spaces [2].

For this study, homelessness was verified at enrolment through direct interviews and assessments of the participant's current living situations. Participants were considered eligible if they reported that they did not have access to a regular, fixed residential dwelling and were living on the streets, pavements and railway/bus stations; under flyovers; in temporary/open spaces; or in other locations not constituting a conventional residence.

Informed written consent was obtained from all eligible patients prior to enrolment. For patients who were unable to consent (e.g., altered sensorium), deferred consent was obtained from the treating team.

Sample size

All eligible patients meeting inclusion criteria were included in the study. Based on a previous census of the ED, approximately two homeless patients visited the ED each month. Over a 15-month data collection period, the expected sample size was approximately 30 patients; however, all eligible homeless patients who visited the ED were enrolled, yielding a final sample size of 48.

Data collection

A structured data collection pro forma was used to record sociodemographic variables, substance use history, the mode of transport to the ED, the type of referral, clinical data (including presenting complaints, triage area, primary survey findings {airway, breathing, circulation, disability and exposure, ABCDE}, clinical examination findings, provisional diagnosis and investigations performed), interdepartmental consultations, total expenditure in the ED, the season of presentation and ED disposition.

Outcomes

The primary outcome is the description of the sociodemographic and clinical profile of homeless patients. Secondary outcomes are (1) ED disposition (discharged, admitted to ward, admitted to ICU, died in ED, LAMA or absconded), (2) ED length of stay, (3) in-ED mortality and (4) ED revisits during the study period.

Statistical analysis

Data were entered in Microsoft Excel (Microsoft Corp., Redmond, WA) and analysed using SPSS version 23.0 (IBM Corp., Armonk, NY). Categorical variables were expressed as frequencies and percentages with 95% confidence intervals (CI). Continuous variables were tested for normality using the Shapiro-Wilk test; normally distributed variables were expressed as mean ± standard deviation (SD) and non-normally distributed variables as median (interquartile range, IQR). This study was exploratory in design, and no prior hypothesis was tested.

Ethics

The study was approved by the Institutional Ethics Committee of AIIMS Rishikesh with letter number AIIMS/IEC/22/453 on 20/10/2022. Written informed consent was obtained from all participants. Data confidentiality was maintained throughout. No conflict of interest exists.

## Results

A total of 48 homeless patients were enrolled over the 18-month study period.

Triage distribution

Triage distribution is summarised in Table 1. The majority of patients presented in critical condition: 32 (66.7%) were triaged to the red (emergency) area, 15 (31.2%) to the yellow (urgent) area and only one (2.1%) to the green (non-urgent) area.

**Table 1:** Distribution of patients by triage area CI: confidence interval

Triage Area	Frequency (n)	Percentage (%)	95% CI
Red (emergency)	32	66.7	51.5%-79.2%
Yellow (urgent)	15	31.2	19.1%-46.4%
Green (non-urgent)	1	2.1	0.1%-12.5%
Total	48	100	-

Sociodemographic profile

The sociodemographic characteristics of the cohort are summarised in Table 2.

**Table 2:** Sociodemographic profile of homeless patients presenting to the emergency department (ED) SD, standard deviation; IQR, interquartile range; CI, confidence interval; INR, Indian rupee

Variable	n (%)	95% CI
Age group (years)		
18-30 (young adult)	10 (20.8%)	11.0%-35.4%
31-60 (adult)	25 (52.1%)	37.4%-66.5%
>60 (elderly)	13 (27.1%)	15.7%-42.1%
Sex		
Male	45 (93.8%)	81.8%-98.4%
Female	3 (6.2%)	1.6%-18.2%
Geographic origin		
Uttarakhand	41 (85.4%)	71.6%-93.5%
Uttar Pradesh	2 (4.2%)	0.7%-15.4%
Punjab	2 (4.2%)	0.7%-15.4%
Jharkhand	1 (2.1%)	0.1%-12.5%
Haryana	1 (2.1%)	0.1%-12.5%
Bihar	1 (2.1%)	0.1%-12.5%
Educational status		
Uneducated	24 (50.0%)	35.4%-64.6%
Graduate	8 (16.7%)	8.0%-30.8%
Pre-primary school	6 (12.5%)	5.2%-25.9%
Secondary school	5 (10.4%)	3.9%-23.4%
Primary school	3 (6.2%)	1.6%-18.2%
High school	2 (4.2%)	0.7%-15.4%
Marital status		
Married	32 (66.7%)	51.5%-79.2%
Unmarried	16 (33.3%)	20.8%-48.5%
Living status before ED arrival		
Staying alone	36 (75.0%)	60.1%-85.9%
Living with others	12 (25.0%)	14.1%-39.9%
Medical insurance type		
Uninsured	39 (81.2%)	66.9%-90.6%
Ayushman Bharat beneficiary	9 (18.8%)	9.4%-33.1%
Annual income (INR)		
Mean (SD)	67,548 (50,603)	-
Median (IQR)	50,000 (35,000-80,000)	-
Range	4,000-200,000	-

Age

Out of the 48 patients, 25 (52.1%) were adults (31-60 years), 13 (27.1%) were elderly (>60 years) and 10 (20.8%) were young adults (18-30 years). In-ED mortality was 1/10 (10.0%) among young adults, 5/25 (20.0%) among adults and 3/13 (23.1%) among elderly patients (Fisher-Freeman-Halton exact p = 0.790). Alcohol use was 3/10 (30.0%), 6/25 (24.0%) and 2/13 (15.4%), respectively (exact p = 0.668). Smoking prevalence differed by age group: 2/10 (20.0%) among young adults, 11/25 (44.0%) among adults and 10/13 (76.9%) among elderly patients (exact p = 0.029; Cramér's V = 0.40), indicating a moderate association between older age and smoking history in this cohort.

Sex

The majority were men (93.8%, n = 45); only three patients (6.2%) were women.

Geographic Origin

Forty-one (85.4%) patients were from Uttarakhand; two (4.2%) patients were from Punjab, two (4.2%) patients were from Uttar Pradesh, one (2.1%) patient was from Jharkhand, one (2.1%) patient was from Haryana and one (2.1%) patient was from Bihar.

Education

Twenty-four (50.0%) were uneducated, and eight (16.7%) had completed graduation. Mean annual income was numerically higher among graduates (Indian rupee {INR} 102,500) than among uneducated patients (INR 33,625). Smoking prevalence was 4/8 (50.0%) among graduates and 15/24 (62.5%) among uneducated patients (Fisher's exact odds ratio {OR} = 0.61; 95% CI, 0.09-4.16; p = 0.684).

Living Situation

Thirty-six (75.0%) were staying alone. Smoking prevalence was 17/36 (47.2%) among patients staying alone versus 6/12 (50.0%) among those living with others (OR = 0.90; 95% CI, 0.20-4.08; p = 1.000); alcohol use was 9/36 (25.0%) versus 2/12 (16.7%), respectively (OR = 1.65; 95% CI, 0.27-18.30; p = 0.705).

Insurance

Thirty-nine (81.2%) patients were uninsured; nine (18.8%) had Ayushman Bharat coverage. Uninsured status was seen in 9/10 (90.0%) young adults, 20/25 (80.0%) adults and 10/13 (76.9%) elderly patients (exact p = 0.791).

Annual Income

Mean (SD) annual income was INR 67,548 (±50,603). The distribution was non-normal (Shapiro-Wilk p < 0.001); median (IQR) was INR 50,000 (35,000-80,000); range was INR 4,000-200,000.

All post hoc subgroup comparisons are summarised in Table 3. Two comparisons reached nominal significance. Smoking prevalence rose monotonically across age groups, from 2/10 (20.0%) in young adults to 11/25 (44.0%) in adults and 10/13 (76.9%) in elderly patients (exact p = 0.029), with a Cramér's V of 0.40 indicating a moderate association, the only effect size of appreciable magnitude in the table. In-ED mortality was lower among smokers than non-smokers (1/23, 4.3%, versus 8/25, 32.0%; OR = 0.10; 95% CI, 0.002-0.87; exact p = 0.024).

**Table 3:** Post hoc subgroup comparisons χ² statistics are shown for descriptive comparability only; all p-values are exact. Odds ratios (OR) are conditional maximum-likelihood estimates with exact 95% confidence intervals (CI). No comparison remains significant after adjustment for multiplicity FFH, Fisher-Freeman-Halton exact test; BH q, Benjamini-Hochberg false discovery rate across all nine comparisons

Comparison	Test	Statistic	Effect Size (95% CI)	Exact p	BH q
Age group versus in-ED mortality	FFH exact	χ² = 0.69	V = 0.12	0.790	0.890
Age group versus alcohol use	FFH exact	χ² = 0.72	V = 0.12	0.668	0.890
Age group versus smoking	FFH exact	χ² = 7.66	V = 0.40	0.029	0.129
Age group versus insurance status	FFH exact	χ² = 0.69	V = 0.12	0.791	0.890
Education versus smoking	Fisher's exact	-	OR = 0.61 (0.09%-4.16%)	0.684	0.890
Smoking versus in-ED mortality	Fisher's exact	-	OR = 0.10 (0.002%-0.87%)	0.024	0.129
Alcohol use versus in-ED mortality	Fisher's exact	-	OR = 0 (0%-1.58%)	0.095	0.284
Living status versus smoking	Fisher's exact	-	OR = 0.90 (0.20%-4.08%)	1.000	1.000
Living status versus alcohol use	Fisher's exact	-	OR = 1.65 (0.27%-18.30%)	0.705	0.890

The remaining seven comparisons were null and uniformly so. Age group was unrelated to in-ED mortality (p = 0.790), alcohol use (p = 0.668) or insurance status (p = 0.791), with Cramér's V of 0.12 in each case, indicating negligible association. Educational attainment was not associated with smoking (OR = 0.61; 95% CI, 0.09-4.16; p = 0.684), and living alone was associated with neither smoking (OR = 0.90; 95% CI, 0.20-4.08; p = 1.000) nor alcohol use (OR = 1.65; 95% CI, 0.27-18.30; p = 0.705). No alcohol user died in the ED (0/11 versus 9/37, 24.3%), but the exact confidence interval for this comparison extends to 1.58 and thus includes the null (p = 0.095).

Neither nominally significant result survived adjustment for multiplicity: across the nine comparisons, both age versus smoking and smoking versus in-ED mortality yielded Benjamini-Hochberg q = 0.129, well above conventional thresholds. All confidence intervals were wide, most spanning one or more orders of magnitude, reflecting the small number of events available for subgroup analysis.

Mode of presentation

Mode of transport is summarised in Table 4: 41 (85.4%) arrived by ambulance, six (12.5%) by public transport and one (2.1%) by private vehicle.

**Table 4:** Mode of prehospital transportation, referral pattern and season of presentation of homeless patients to the emergency department (ED) CI: confidence interval

Variable	n (%)	95% CI
Mode of prehospital transportation		
Ambulance	41 (85.4%)	71.6%-93.5%
Public transport	6 (12.5%)	5.2%-25.9%
Private vehicle	1 (2.1%)	0.1%-12.5%
Type of ED presentation		
Referred from another healthcare facility	32 (66.7%)	51.5%-79.2%
Direct visit	16 (33.3%)	20.8%-48.5%
Season of presentation		
Autumn	16 (33.3%)	20.8%-48.5%
Summer	15 (31.2%)	19.1%-46.4%
Spring	13 (27.1%)	15.7%-42.1%
Winter	4 (8.3%)	2.7%-20.9%

The type of ED presentation is summarised in Table 4: 32 (66.7%) were referred from another healthcare facility; 16 (33.3%) had a direct visit.

The season of presentation is summarised in Table 4: 16 (33.3%) patients presented in autumn, 15 (31.2%) patients presented in summer, 13 (27.1%) patients presented in spring and four (8.3%) patients presented in winter.

Clinical profile

The clinical profile is summarised in Table 5: The most common chief complaints were abdominal symptoms in 11 (22.9%) patients, followed by dyspnoea in nine (18.8%) patients, trauma in eight (16.7%) patients and neurological symptoms in seven (14.6%) patients, and four (8.3%) patients were brought dead. Chest pain and poisoning each accounted for three (6.2%) presentations, while hanging, bite/stings and limb pain each accounted for one (2.1%) presentation.

**Table 5:** Clinical presentation and co-morbidities of homeless patients in the emergency department AMS, altered mental status; CAD, coronary artery disease; CLD, chronic liver disease; COPD, chronic obstructive pulmonary disease; CVA, cerebrovascular accident; CI, confidence interval

Variable	n (%)	95% CI
Presenting complaint		
Abdominal symptoms (pain, jaundice, nausea and vomiting)	11 (22.9%)	12.5%-37.7%
Dyspnoea	9 (18.8%)	9.4%-33.1%
Trauma	8 (16.7%)	8.0%-30.8%
Neurological symptoms (AMS and seizures)	7 (14.6%)	6.5%-28.4%
Brought dead	4 (8.3%)	2.7%-20.9%
Chest pain	3 (6.2%)	1.6%-18.2%
Poisoning	3 (6.2%)	1.6%-18.2%
Bite/stings	1 (2.1%)	0.1%-12.5%
Hanging	1 (2.1%)	0.1%-12.5%
Limb pain	1 (2.1%)	0.1%-12.5%
Co-morbidities (present)		
Hypertension	6 (12.5%)	5.2%-25.9%
Others (CAD, CLD, etc.)	6 (12.5%)	5.2%-25.9%
COPD	4 (8.3%)	2.7%-20.9%
Diabetes mellitus	1 (2.1%)	0.1%-12.5%
Chronic kidney disease	1 (2.1%)	0.1%-12.5%
CVA	1 (2.1%)	0.1%-12.5%

Co-morbidities are summarised in Table 5: hypertension was present in six patients (12.5%), chronic obstructive pulmonary disease (COPD) in four (8.3%) and diabetes mellitus, chronic kidney disease and cerebrovascular accident in one patient each (2.1%). A further six patients (12.5%) had other co-morbidities, each occurring once (including coronary artery disease and chronic liver disease).

Provisional diagnoses

The provisional diagnosis is summarised in Table 6: COPD was the most frequent diagnosis in six (12.5%) patients, followed by head injury in five (10.4%) patients, brought dead in four (8.3%) patients, decompensated chronic liver disease (DCLD) in four (8.3%) patients and poisoning in three (6.2%) patients. A wide range of other diagnoses included meningitis, status epilepticus, acute heart failure, obstructive uropathy, dengue, liver abscess, malignancy and self-harm (Table 6).

**Table 6:** Distribution by provisional diagnosis COPD, chronic obstructive pulmonary disease; CLD, chronic liver disease; LRTI, lower respiratory tract infection

Diagnosis	n	%
COPD	6	12.5
Head Injury	5	10.4
Brought dead	4	8.3
Decompensated CLD	4	8.3
Poisoning	3	6.2
Altered mental status	2	4.2
Coronary artery disease	2	4.2
Fracture/soft tissue injury	2	4.2
LRTI	2	4.2
Obstructive uropathy	2	4.2
Others	16	33.3

Investigations and consultations

Point-of-care ultrasound (POCUS) (lung scan, echo and inferior vena cava {IVC} collapsibility), ECG and chest X-ray were done when clinically indicated based on presenting complaint and treating physician judgement. Standard haematological and biochemical investigations were obtained where clinically indicated.

Interdepartmental consultations

Consultations were most commonly sought from general medicine in 21 (43.8%) patients, neurology in 10 (20.8%) patients, pulmonary medicine in eight (16.7%) patients, neurosurgery in eight (16.7%) patients, orthopaedics and trauma surgery in eight (16.7%) patients, psychiatry in eight (16.7%) patients, general surgery in seven (14.6%) patients, nephrology in seven (14.6%) patients, gastroenterology in four (8.3%) patients, urology in three (6.2%) patients and cardiology in three (6.2%) patients.

Total expenditure in ED

Mean (SD) expenditure was INR 3,923 (±2,716); median (IQR) was INR 3,000 (2,000-5,000). All expenses were met through free billing (government/hospital funds) or the Ayushman Bharat scheme, as most patients were either uninsured or scheme beneficiaries.

ED outcomes

ED outcomes are summarised in Table 7: 25 (52.1%) patients were discharged from the ED, nine (18.8%) patients died in the ED, five (10.4%) patients left against medical advice (LAMA), four (8.3%) patients were admitted to the ICU, four (8.3%) patients were admitted to the ward and one (2.1%) patient absconded (brought dead patients were included in the died in ED section) (Table 7).

**Table 7:** Emergency department (ED) disposition, length of stay and revisits among homeless patients CI, confidence interval; SD, standard deviation; IQR, interquartile range

Variable	n (%)/Value	95% CI
ED disposition		
Discharged from ED	25 (52.1%)	37.4%-66.5%
Died in ED	9 (18.8%)	9.4%-33.1%
Left against medical advice (LAMA)	5 (10.4%)	3.9%-23.4%
Admitted to ICU	4 (8.3%)	2.7%-20.9%
Admitted to ward	4 (8.3%)	2.7%-20.9%
Absconded from ED	1 (2.1%)	0.1%-12.5%
ED length of stay (days)		
Mean (SD)	2.77 (2.01)	-
Median (IQR)	2 (2-3)	-
Range	1-12	-
ED revisits during study period (excluding patients who died in the ED or were brought dead, n = 9)		
No revisit	25 (64.1%)	-
1 revisit	10 (25.6%)	-
3 revisits	4 (10.3%)	-

ED length of stay is summarised in Table 7: mean (SD) was 2.77 days (±2.01). Median (IQR) was two days (2-3), with a range of 1-12 days. Brought dead patients were excluded during ED length of stay calculation.

ED revisits are summarised in Table 7: 14 (35.9%) of 39 patients had ED revisits during the study period (excluding patients who died in the ED or were brought dead, n = 9); 10 (25.6%) patients had one revisit, and four (10.3%) patients had three revisits. Revisits were captured by using unique hospital identification number (UHID) against the ED registration log for the full study period.

## Discussion

This prospective observational study is one of the first from India to systematically characterise the sociodemographic and clinical profile of 48 homeless patients presenting to a tertiary care emergency department over 18 months. The principal findings are descriptive: the cohort was overwhelmingly male, uninsured and locally resident; was presented predominantly by ambulance and in critical condition; and experienced substantial in-ED mortality. Subgroup comparisons were exploratory, and no association survived correction for multiple testing; they are reported to inform future hypothesis-driven work rather than to support inference.

The predominance of male patients (45, 93.8%) is consistent with global literature. Tadros et al. [19] reported 72.3% men, and Burak et al. [20] and Little and Watson [21] reported comparable male predominance. The low representation of women likely reflects gender differences in stigma and vulnerability that affect both the visibility and the reporting of homeless women, rather than a true absence of need; women experiencing homelessness may preferentially seek shelter-based or informal care.

Most patients (25, 52.1%) were in the 31-60-year age group, with 13 (27.1%) elderly (>60 years). Age group was not associated with in-ED mortality (exact p = 0.790), alcohol use (exact p = 0.668) or insurance status (exact p = 0.791), with Cramér's V of 0.12 for each, indicating negligible association. These null results should be attributed to limited power as much as to a true absence of effect: With nine deaths and 11 alcohol users across three age strata, the study can exclude only very large effects, and larger multi-centre studies are needed. Smoking prevalence did differ by age group, rising from 2/10 (20.0%) among young adults to 11/25 (44.0%) among adults and 10/13 (76.9%) among elderly patients (Fisher-Freeman-Halton exact p = 0.029; Cramér's V = 0.40). This was the only association of appreciable magnitude in the cohort and is consistent with cumulative lifetime exposure during prolonged street residence, although it does not survive correction for multiple comparisons (Benjamini-Hochberg q = 0.129) and cross-sectional data cannot separate a cohort effect from differential survival.

The substantial proportion of elderly patients carries clinical implications, as older homeless individuals bear a higher burden of chronic disease and worse outcomes. This aligns with Brown and Steinman's finding that older homeless patients have more severe medical presentations [6]. In our cohort, in-ED mortality was numerically higher in older groups (1/10, 10.0%, in young adults; 5/25, 20.0%, in adults; and 3/13, 23.1%, in the elderly), but this gradient was not statistically significant (exact p = 0.790; Cramér's V = 0.12) and is compatible with chance at this sample size. It is reported as a descriptive observation rather than as evidence of an age-mortality relationship.

Thirty-two patients (66.7%) were triaged to the red (immediate/emergency) area, indicating that homeless patients present critically ill. This high acuity is consistent with barriers to earlier ambulatory care that have been described in homeless populations, although our single-centre descriptive design cannot establish the timing or causality of presentation.

The ambulance utilisation rate of 41/48 (85.4%) far exceeds the 51% reported by Pearson et al. in the USA, suggesting that homeless patients in this setting reach the ED largely passively, transported by others rather than self-presenting [10]. That 32 patients (66.7%) were referred from another healthcare facility rather than presenting directly further suggests that a substantial proportion of patients are transferred after initial contact elsewhere. This observation is compatible with capacity constraints at primary and secondary levels, but our data cannot quantify those constraints or attribute causality.

Abdominal symptoms (11, 22.9%) were the most common presenting complaint, followed by dyspnoea (nine, 18.8%) and trauma (eight, 16.7%). Unlike Western studies, in which substance-related and psychiatric presentations predominate, our cohort showed a higher burden of medical illness, COPD, chronic liver disease and infectious disease, reflecting disease epidemiology and environmental exposures particular to the Indian homeless population. Psychiatry consultation was nonetheless sought in eight patients (16.7%), so psychiatric morbidity is present but was not the leading reason for attendance.

The in-ED mortality rate of 9/48 (18.8%) is notably high and reflects the severity of illness at presentation. A global systematic review by Vohra et al. documented high adverse outcomes among homeless ED patients [16]; our rate sits at the upper end of reported ranges, likely reflecting the combination of late presentation, high disease burden and resource constraints. That four patients (8.3%) were brought dead further underscores the critical nature of these presentations.

Among subgroup comparisons, in-ED mortality was lower among smokers (1/23, 4.3%) than non-smokers (8/25, 32.0%; Fisher's exact OR = 0.10; 95% CI, 0.002-0.87; p = 0.024). This must not be read as a protective effect of smoking, and three considerations argue against any causal interpretation. First, the estimate rests on a single death among 23 smokers, and its confidence interval spans more than two orders of magnitude. Second, it does not survive correction for multiplicity across the nine post hoc comparisons reported in Table 3 (Benjamini-Hochberg q = 0.129). Third, at least two noncausal mechanisms could generate it: confounding by presentation severity, if non-smokers presented with more acute illness, a greater proportion brought dead or in advanced organ failure, than the older, chronic-disease-burdened smoking subgroup, and collider stratification, since both smoking status and survival to ED registration depend on the pathway by which a homeless individual reaches hospital. We regard this finding as an artefact of sparse data and report it for completeness rather than as a result.

No alcohol user died in the emergency department (0/11) compared with 9/37 (24.3%) of nonusers. Because one cell is zero, the conditional maximum-likelihood odds ratio is 0 with an exact 95% confidence interval of 0-1.58. This analysis is uninformative rather than negative: It has essentially no power to exclude an effect in either direction, and it should not be presented as a parallel to, or corroboration of, the smoking finding.

Thirty-nine patients (81.2%) were uninsured, and only nine (18.8%) were covered under Ayushman Bharat, a figure that likely underrepresents true eligibility in this population given the documentation requirements for enrolment. Uninsured status did not differ across age groups (exact p = 0.791). This is consistent with literature documenting that the absence of insurance drives delayed healthcare-seeking and adverse outcomes among homeless populations and identifies scheme enrolment at the point of ED contact as a tractable intervention.

The median ED length of stay, excluding patients brought dead, was two days (IQR, 2-3; mean, 2.77 days; SD, 2.01), substantially longer than the 2.6-7.5 hours reported by Pearson et al. [10]. This difference is of an order that reflects a structural rather than a clinical distinction: prolonged stay here represents boarding driven by the practical impossibility of discharging a homeless patient without destination, caretaker or follow-up arrangement, compounded by medical complexity. Mean expenditure was INR 3,923 (SD: 2,716) per patient, borne entirely by the hospital or the Ayushman Bharat scheme, highlighting the financial burden carried by the public health system.

Fourteen of the 39 patients who survived the index visit (35.9%, 95% CI: 21.2%-52.8%) had at least one ED revisit during the study period, and four (10.3%) returned three times. Restricting the denominator to patients actually at risk of revisit gives a higher and more interpretable estimate than one computed across the entire cohort. Doran et al. reported that 53.9% of hospital readmissions occurred within one week of discharge [8]; our data reinforce the pattern of recurrent utilisation driven by absent continuity of care, social support and stable housing. Direct comparison with fixed-interval revisit rates is limited, however, because our observation window varied by enrolment date rather than being anchored at a fixed interval after discharge, and revisits were captured only within this centre.

Clinical implications

Our findings highlight the urgent need for (1) dedicated ED protocols for homeless patients, including social worker engagement at the time of triage; (2) integration with Ayushman Bharat and other government welfare schemes at the point of ED contact; (3) outreach and mobile health programmes targeting homeless communities for early disease detection; (4) multiagency post-discharge planning to prevent the revolving-door pattern of ED utilisation; and (5) the training of ED staff to recognise and address the unique needs of this population.

Limitations

This study may have insufficient sample size to determine the actual validity of these results. Further studies with bigger sample sizes are needed to determine more valid results.

This was a single-centre, prospective, descriptive observational study without a concurrent comparison group of non-homeless patients.

This study did not include the post-discharge follow-up of homeless patients.

## Conclusions

Homeless patients presenting to the ED at AIIMS Rishikesh are predominantly men, middle-aged and uninsured and arrive critically ill by ambulance, usually referred from other facilities. They carry a high burden of chronic disease, including COPD, chronic liver disease and trauma and frequently present with advanced or critical illness. The in-ED mortality rate was 18.8%, with a notable proportion brought dead. ED length of stay was longer than general populations, and revisit rates were high.

These findings underscore the need for targeted, integrated care strategies within the emergency department, including systematic social assessment at triage, linkage with government welfare schemes and structured post-discharge planning. This study provides foundational data for future multi-centre research and evidence-based policymaking for homeless health in India.
